# The prognostic value of extracranial vascular characteristics on procedural duration and revascularization success in endovascularly treated acute ischemic stroke patients

**DOI:** 10.1177/23969873211067662

**Published:** 2022-02-08

**Authors:** Ghislaine Holswilder, Maaike PME Stuart, Tine Dompeling, Nyika D Kruyt, Jelle J Goeman, Aad van der Lugt, Wouter J Schonewille, Geert J Lycklama à Nijeholt, Charles BLM Majoie, Lonneke SF Yo, Frederick JA Meijer, Henk A Marquering, Marieke JH Wermer, Marianne AA van Walderveen, on behalf of the MR CLEAN Registry investigators

**Affiliations:** 1 Department of Radiology, 4501 Leiden University Medical Center, Leiden, Netherlands; 2 Department of Neurology, 4501 Leiden University Medical Center, Leiden, Netherlands; 3 Department of Biomedical Data Sciences, 4501 Leiden University Medical Center, Leiden, Netherlands; 4 Department of Radiology, 6993 Erasmus MC University Medical Center, Rotterdam, Netherlands; 5 Department of Neurology, 6028 Sint Antonius Hospital, Nieuwegein, Netherlands; 6 Department of Radiology, 2901 Haaglanden MC, the Hague, Netherlands; 7 Department of Radiology and Nuclear Medicine, 522567 Amsterdam University Medical Center, University of Amsterdam, Amsterdam, Netherlands; 8 Department of Radiology, 3168 Catharina Hospital, Eindhoven, Netherlands; 9 Department of Radiology and Nuclear Medicine, 6034 Radboud University Medical Center, Nijmegen, Netherlands

**Keywords:** Stroke, Endovascular treatment, aortic arch, carotid arteries, tortuosity

## Abstract

**Introduction:**

Vascular anatomy might affect endovascular treatment success in acute ischemic stroke patients with large vessel occlusion. We investigated the prognostic value of extracranial vascular characteristics on procedural time and revascularization success in patients with large vessel occlusion in the anterior cerebral circulation.

**Patients and methods:**

We included 828 patients endovascularly treated within 6.5 hours of symptom onset from the Dutch MR CLEAN-Registry. We evaluated aortic arch configuration, stenosis and tortuosity of supra-aortic arteries, and internal carotid arteries (ICAs) on pre-intervention CTA. We constructed logistic prediction models for outcome variables procedural duration (≥60 minutes) and non-successful revascularization (extended thrombolysis in cerebral infarction (eTICI) of 0–2A) using baseline characteristics and assessed the effect of extracranial vascular characteristics on model performance.

**Results:**

Cervical ICA tortuosity and stenosis ≥99% improved prediction of long procedural duration compared with baseline characteristics from area under the curve of 0.61 (95% CI: 0.57–0.65) to 0.66 (95% CI: 0.62–0.70) (*P* < 0.001). Cervical ICA tortuosity was significantly associated with non-successful recanalization. Prediction of non-successful revascularization did not improve after including aortic arch elongation, acute take-off angle, aortic variant, origin stenosis of supra-aortic arteries, and cervical ICA tortuosity, with an area under the curve of 0.63 (95% CI: 0.59–0.67) compared with 0.59 (95% CI: 0.55–0.63) (*P* = 0.11).

**Conclusion:**

Extracranial vascular characteristics have additional prognostic value for procedural duration, but not for revascularization success, compared with baseline characteristics. Performance of both prediction models is limited in patients treated for large vessel occlusion.

## Introduction

Endovascular treatment (EVT) is highly effective in patients with acute ischemic stroke with large vessel occlusion (LVO) in the anterior circulation. Patient recovery after EVT is closely related to successful revascularization as this is associated with improved functional outcome.^1^ Also, time to revascularization is important as the probability of functional independence decreases 7.7% with every hour delay from symptom onset to revascularization.^2^ The goal of EVT is, therefore, to achieve the highest degree of revascularization with the shortest possible delay.^3^ Current recommendations state that procedural time should not exceed 60 minutes and the target should be successful recanalization defined as restored antegrade flow of ≥50% of the territory of the previously occluded artery.^4^

Recent studies demonstrated that unfavorable extracranial vascular anatomy, including complex configuration of the aortic arch and tortuosity of the supra-aortic vessels, prolongs procedural duration,^5–7^ and negatively influences revascularization success.^5,7,8^ However, these studies were performed with small sample sizes leaving uncertainty about the true association between vascular characteristics and procedural duration and revascularization success, especially in addition to baseline predictors. Knowledge of these associations would be of value in clinical practice, especially when this information can be derived from pre-intervention CT angiography (CTA), to better direct arterial access approaches for EVT of LVO ischemic stroke patients.

We aimed to associate extracranial vascular characteristics on pre-intervention CTA with procedural duration and revascularization success and assess their added value in prediction models compared with baseline predictors in a large population of patients with LVO treated with EVT.

### Patients and methods

**Figure 1.**
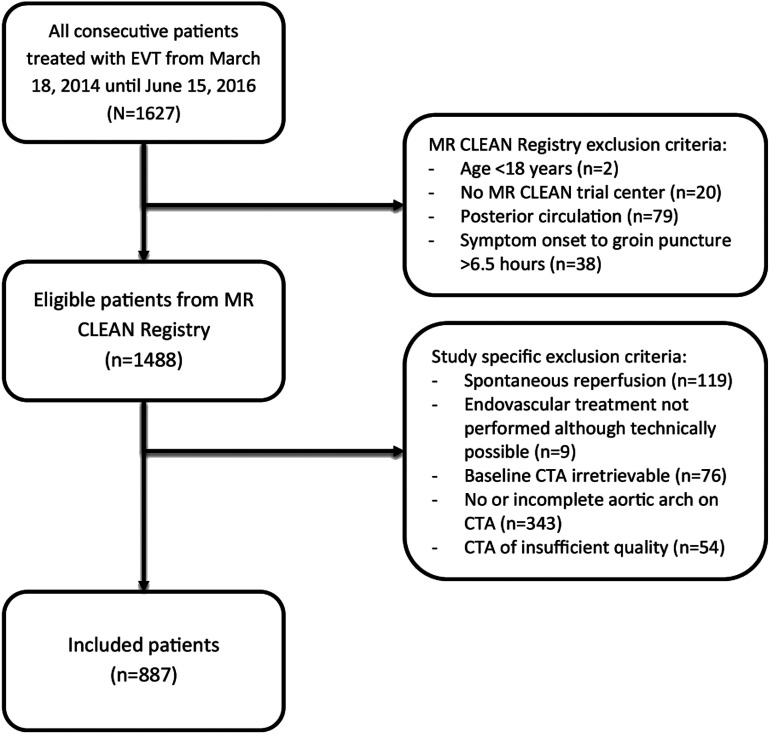
**Inclusion flowchart.** From the MR CLEAN Registry, 1488 patients were available for analysis of which 601 patients were excluded based on study specific criteria, leading to the final inclusion of 887 patients. CTA: CT angiography; EVT: endovascular treatment.

Patients were included from the Multicenter Randomized Controlled Trial of Endovascular Treatment for Acute Ischemic Stroke in the Netherlands (MR CLEAN) Registry from March 2014 until June 2016.^9^ The MR CLEAN Registry was an observational registry containing prospectively recorded data from 16 centers in the Netherlands that perform EVT. Patients were included when they were ≥18 years, had a diagnosis of acute ischemic stroke due to an LVO in the anterior cerebral circulation, and received EVT within 6.5 hours of symptom onset. Baseline clinical data, including patient history and stroke characteristics (e.g., stroke risk factors, National Institutes of Health Stroke Scale (NIHSS)) were available.^9^ Baseline imaging (non-contrast brain CT, CTA of the aorta, cervical, and cerebral vessels) and follow-up radiological data (post-intervention extended thrombolysis in cerebral infarction (eTICI)) were assessed by an imaging core laboratory.^9^ A detailed description of the registry procedures is published elsewhere.^9^ For this study, patients were excluded when EVT was not performed because the LVO was no longer present on digital subtraction angiography (DSA) (e.g., because the thrombus had dissolved, either spontaneously or because of IV thrombolysis treatment, or had migrated distally) or if the intervention was technically possible but stopped for other reasons (e.g., vessel perforation during catheterization). Furthermore, patients were excluded when baseline CTA was not available, did not include the aortic arch and/or cervical vessels, or was of insufficient quality. (Figure 1).

### CTA analysis

For this study, CTA of aortic arch, supra-aortic, and internal carotid arteries (ICAs) were retrospectively analyzed (Picture Archiving and Communicating System, Sectra IDS7 18.2, Linköping, Sweden) by trained students (MPMES and TD) under supervision of MAAW (neuroradiologist with >20 years of experience in CTA analysis). Interobserver agreement of vascular characteristics was determined in a random selection of 100 patients with Cohen’s kappa (κ); values 0.41–0.60 were considered moderate agreement, 0.61–0.80 good agreement, and >0.80 excellent agreement. All observers were blinded to clinical information except for patients’ sex, age, and side of intracranial occlusion. Window and level settings for CTA analysis were standardized at W:750, C:200.^10^ Based on the literature, the following vascular characteristics were selected: aortic arch variants,^11^ aortic arch elongation,^12,13^ tortuosity^12–15^ and stenosis^12^ of supra-aortic arteries (innominate artery (IA), common carotid arteries (CCA)), and tortuosity^12–15^ and stenosis^12^ of the ICA.

Aortic arch variants were defined as: type A) the IA, left CCA, and left subclavian artery branch directly from the aortic arch; type B) the IA and left CCA share a common origin; or type C) the left CCA branches from the IA.^11^ Aortic arch elongation was assessed according to previous studies and divided into three types (aortic arch type I, II, or III; a more detailed description is provided in Figure S1A of the Supplementary material).^12,13^

The take-off angle from the aorta to the supra-aortic artery ipsilateral to the side of the LVO, was measured in an adjusted projection to show the maximal angle of the aortic arch and the origin of the supra-aortic artery.^13^ (Figure S1B and S1C). A normal angle was defined as a 90° angle and a difficult take-off angle was defined, based on expert opinion, as an acute angle measuring ≥135°.

Tortuosity of supra-aortic arteries (IA/CCA) and ICA, ipsilateral to the side of the intracranial occlusion, was evaluated by assessing the angulation of these vessels.^13–15^ Tortuosity was defined as the presence of at least one angle measuring ≥90°; in addition, presence of two angles or more measuring ≥90° was assessed. (Figure S1D).

Stenosis was assessed for IA/CCA, cervical, and intracranial ICA ipsilateral to the side of the intracranial occlusion. Stenosis was visually determined at the origin of the supra-aortic arteries (IA/CCA) and dichotomized into <50% and ≥50%.^12^ Presence of stenosis of the cervical ICA was measured according to the North American Symptomatic Carotid Endarterectomy Trial (NASCET) criteria and dichotomized into <99% and ≥99% (including occlusion).^16^ Intracranial ICA stenosis was visually determined and dichotomized into <50% and ≥50%.

### Outcome measures

Procedural duration was defined as the duration of EVT from groin puncture to removal of the catheter sheath and dichotomized into <60 minutes and ≥60 minutes, based on current recommendations that procedure time should not exceed 60 minutes.^4^ Procedural duration cannot be defined in patients in whom the occlusion site could not be reached by transfemoral approach; these patients were therefore not included in the analysis for procedural duration. Non-successful revascularization after EVT was defined as eTICI grade 0–2A on the final DSA run.^1,17^ To grade eTICI ≥2B, the final run had to contain both anteroposterior and lateral views; presence of only one view resulted in a maximal grade of 2A.

### Statistical analysis

Baseline characteristics were reported as number (%), mean (standard deviation; SD), or median (interquartile range; IQR). To analyze the effect of vascular characteristics on pre-intervention CTA on outcome parameters, we first assessed associations by logistic regression and adjusted for covariates known prior to EVT. For procedural duration, we adjusted for age,^12,18^ hypertension,^19^ and clot burden score (CBS)^20^; for non-successful revascularization, we adjusted for age, hypertension, CBS, intravenous thrombolysis (IVT),^18^ pre-stroke eTICI, and collateral score.^21^ Multiple imputation with 10 imputations was performed to obtain unbiased analyses, using the Markov chain Monte Carlo method (variables are described in Table S1).^22^

Second, we constructed logistic prediction models to assess which combination of vascular characteristics is associated with the outcome parameters. We started with baseline prediction models with covariates used for adjustments. We developed final prediction models using backward selection of vascular characteristics; variables were kept in the model when the effect in the model showed a p-value < 0.20. Finally, performance of the final models was assessed using the receiver operating characteristic (ROC) curve and area under the curves (AUC) (pooled with Rubin’s Rule). Whether final models had significantly increased goodness-of-fit compared with baseline models was assessed with the likelihood ratio test (pooled for all imputations).

## Results

We included 887 patients (mean age: 69 years, 52% men) (Figure 1). Baseline characteristics for included and excluded patients are shown in Table S2 of the Supplementary material. The occlusion site was not reached via the transfemoral approach in 59 patients (7%). Procedural duration ≥60 minutes was present in 456 patients (61%) and revascularization after EVT was non-successful (eTICI 0–2A) in 395 patients (45%) (Table 1); in 84% of all patients, DSA included both anteroposterior and lateral views. Baseline characteristics for the groups sorted by outcome measures are shown in Table 1.

**Table 1.** Baseline and outcome characteristics of the total population (N = 887), divided by procedural duration (≥60 vs. <60 minutes) (n = 745) and by revascularization success (eTICI 0–2A vs. eTICI 2B–3) (n = 877).

Patient characteristic	Total N = 887	≥60 minutes n = 456	<60 minutes n = 289	eTICI 0–2A n = 395	eTICI 2B–3 n = 482
Age (mean, SD)	68.7 (14.4)	68.5 (14.1)	67.6 (15.2)	69.7 (14.2)	67.9 (14.5)
Men (n, %)	461 (52%)	245 (54%)	151 (51%)	203 (51%)	254 (53%)
Previous stroke (n, %)	159 (18%)	66 (15%)	63 (21%)	69 (18%)	87 (18%)
Diabetes mellitus (n, %)	136 (15%)	67 (15%)	43 (15%)	61 (16%)	73 (15%)
Hypertension (n, %)	446 (51%)	240 (54%)	138 (47%)	204 (52%)	239 (50%)
Atrial fibrillation (n, %)	202 (23%)	100 (22%)	79 (27%)	98 (25%)	103 (22%)
Current smoking (n, %)	214 (24%)	116 (26%)	70 (24%)	90 (23%)	121 (25%)
Pre-mRS ≥1 (n, %)	286 (33%)	149 (33%)	98 (33%)	133 (34%)	150 (32%)
NIHSS at baseline (median, IQR)	16 (12–20)	16 (12–20)	15 (12–19)	16 (12–20)	15 (11–19)
**Radiological characteristics**					
Pre-intervention eTICI ≥1 (n, %)	83 (10%)	43 (10%)	29 (11%)	33 (9%)	49 (11%)
Collateral score ≥50% (n, %)	512 (60%)	256 (59%)	183 (64%)	209 (55%)	296 (64%)
ASPECTS ≤7 (n, %)	256 (29%)	144 (32%)	79 (27%)	117 (30%)	137 (29%)
CBS ≤7 (n, %)	548 (72%)	284 (73%)	176 (69%)	241 (72%)	300 (72%)
**Intervention characteristics**					
IVT administered (n, %)	683 (77%)	352 (77%)	222 (75%)	298 (75%)	376 (78%)
EVT under general anesthesia (n, %)	231 (27%)	121 (28%)	89 (31%)	79 (22%)	150 (32%)
Symptom onset to groin puncture, min (median, IQR)	210 (159–265)	207 (160–260)	210 (155–261)	214 (159–275)	200 (160–255)
**Outcome characteristics**					
Procedural duration, min (median, IQR)	65 (45–90)	85 (70–105)	40 (31–50)	80 (60–105)	55 (37–75)
mRS after 3 months 3–6	504 (59%)	290 (66%)	131 (45%)	287 (77%)	212 (45%)
eTICI 0–2A	395 (45%)	239 (53%)	58 (20%)	-	-

ASPECTS: Alberta Stroke Program Early CT Score; CBS: clot burden score; eTICI: extended thrombolysis in cerebral infarction; EVT: endovascular treatment; IQR: interquartile range; IVT: intravenous thrombolysis; mRS: modified Rankin Scale; NIHSS: National Institutes of Health Stroke Scale; SD: standard deviation.

Numbers might not add up due to missing values.

### Vascular characteristics

Tortuosity of the cervical ICA (adjusted odds ratio (aOR): 1.9, 95% CI: 1.4–2.7) was significantly associated with procedural duration after adjustment for baseline characteristics (Table 2). All patients with IA/CCA origin stenosis ≥50% and 59% of patients without origin stenosis had a procedural duration ≥60 minutes (Fisher’s exact test *P* = 0.01) (Supplementary material). In addition, cervical ICA stenosis ≥99% was significantly associated with procedural duration ≥60 minutes (aOR: 3.0, 95% CI: 1.6–5.9). Tortuosity of the cervical ICA (aOR: 1.5, 95% CI: 1.1–2.0) was significantly associated with non-successful revascularization (eTICI 0–2A) after adjustment for baseline characteristics. (Table 2, Supplementary material). Interobserver agreement was excellent for aortic variant (κ: 0.90) and acute take-off angle ≥135° (κ: 0.85), good for aortic arch elongation (κ: 0.79) and tortuosity of at least one angle measuring ≥90° (κ: 0.73), and moderate for intracranial ICA stenosis ≥50% (κ: 0.45) and cervical ICA stenosis ≥99% (κ: 0.58).

**Table 2.** Odds ratios of procedural duration ≥60 minutes (n = 828) and non-successful revascularization (eTICI 0–2A) (n = 887) in the presence versus absence of extracranial vascular characteristics.

	Duration ≥60 minutes		eTICI 0–2A	
	Unadjusted OR (95% CI)	Adjusted OR (95% CI)	Unadjusted OR (95% CI)	Adjusted OR (95% CI)
**Aortic arch elongation**				
Type II vs. I	1.1 (0.7–1.6)	1.1 (0.7–1.6)	0.9 (0.7–1.3)	0.8 (0.6–1.2)
Type III vs. I	1.1 (0.7–1.8)	1.1 (0.7–2.0)	1.2 (0.8–1.9)	1.0 (0.6–1.6)
Take-off angle ≥135°	1.4 (0.8–2.5)	1.5 (0.8–2.7)	1.3 (0.8–2.2)	1.4 (0.8–2.3)
**Aortic variants**				
Variant B vs. A	1.1 (0.7–1.7)	1.1 (0.7–1.8)	0.8 (0.6–1.2)	0.9 (0.6–1.3)
Variant C vs. A	1.0 (0.6–1.7)	1.0 (0.6–1.7)	1.5 (0.9–2.3)	1.5 (0.9–2.3)
**Tortuosity right IA and CCA, or left CCA** ^b **,** c^				
Presence of ≥1 angles ≥90°	1.2 (0.9–1.6)	1.1 (0.8–1.5)	1.3 (1.0–1.8)^a^	1.3 (0.9–1.7)
Presence of ≥2 angles ≥90°	1.1 (0.7–1.6)	1.0 (0.6–1.6)	1.3 (0.9–2.0)	1.3 (0.8–1.9)
**Tortuosity cervical ICA** ^b^				
Presence of ≥1 angles ≥90°	2.0 (1.5–2.8)^a^	1.9 (1.4–2.7)^a^	1.6 (1.2–2.1)^a^	1.5 (1.1–2.0)^a^
Presence of ≥2 angles ≥90°	2.0 (1.3–3.1)^a^	2.0 (1.3–3.0)^a^	1.4 (1.0–2.0)^a^	1.4 (0.9–1.9)
**Vessel stenosis**				
IA/CCA origin stenosis ≥50%^d^	-	-	3.1 (0.7–13.7)	3.1 (0.7–13.3)
ICA stenosis ≥99%	3.3 (1.7–6.3)^a^	3.0 (1.6–5.9)^a^	1.3 (0.8–2.1)	1.3 (0.8–2.2)
Intracranial ICA stenosis †≥50%	1.1 (0.7–1.6)	0.9 (0.6–1.4)	1.4 (0.9–2.1)	1.3 (0.9–1.9)

CCA: common carotid arteries; eTICI: extended thrombolysis in cerebral infarction; IA: innominate artery; ICA: internal carotid artery; OR: odds ratio.

Numbers might not add up due to missing values. ORs were adjusted for age, hypertension and CBS for procedural duration, and—in addition to these—for intravenous thrombolysis, pre-stroke eTICI and collateral score for non-successful revascularization.

^a^Indicates significant OR.

^b^The IA and left or right CCA were measured ipsilateral to the side of the intracranial vessel occlusion.

^c^Presence of ≥1 angles ≥90° was compared with no angle ≥90°; presence of ≥2 angles ≥90° was compared with no or 1 angle ≥90°.

^d^(adjusted) OR could not be calculated for procedural duration; Fisher’s exact test resulted in *P* = 0.01.

### Prediction models and ROC analyses

**Figure 2.**
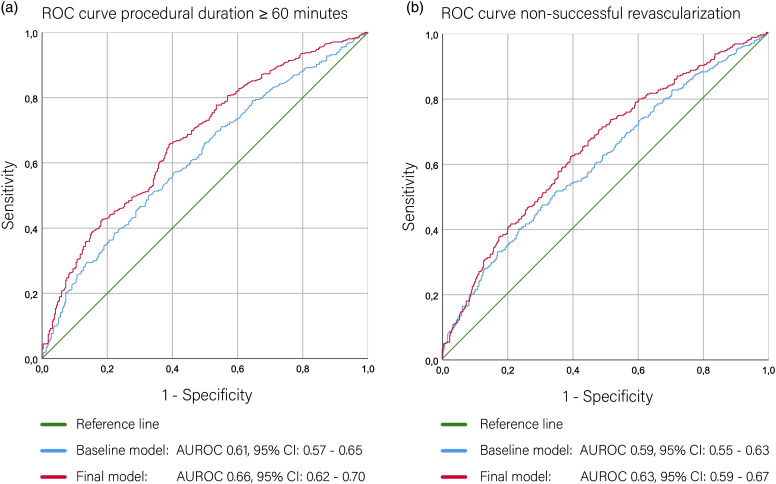
**Receiver operating characteristic (ROC) curves of the baseline model and final model for (a) prediction of procedural duration ≥60 minutes and (b) non-successful revascularization (eTICI 0–2A).** ROC curves represent averaged curves and area under the curve (AUC) values represent pooled AUC’s of all 10 imputations. Baseline models are indicated by blue lines and final models with red lines.

The baseline prediction model of procedural duration ≥60 minutes with age, hypertension, and CBS resulted in an AUC of 0.61 (95% CI: 0.57–0.65). After backward regression, the following characteristics remained in the final model: tortuosity of the cervical ICA and cervical ICA stenosis ≥99%. This final model had an AUC of 0.66 (95% CI: 0.62–0.70). (Figure 2A). Regression coefficients and intercept are shown in Table S4 of the Supplementary material. The likelihood ratio test indicated a significant difference in model performance between the final and baseline model (*P* < 0.001).

Baseline prediction model of non-successful revascularization (eTICI 0–2A) with age, hypertension, IVT, collateral score, CBS, and eTICI prior to EVT treatment resulted in an AUC of 0.59 (95% CI: 0.55–0.63). Following backward selection, the following characteristics remained in the final model: acute take-off angle ≥135°, aortic variant, the presence of IA/CCA origin stenosis ≥50%, tortuosity of the IA/CCA, tortuosity of the cervical ICA, and cervical ICA stenosis ≥99%. This final model had an AUC of 0.63 (95% CI: 0.59–0.67). (Figure 2B). Regression coefficients and intercept are shown in Table S5 of the Supplementary material. The likelihood ratio test indicated no difference in model performance between the final and baseline model (*P* = 0.27).

## Discussion

We showed that tortuosity of the cervical ICA was associated with long procedural duration (≥60 minutes) and non-successful revascularization in a large cohort of consecutive acute anterior circulation ischemic stroke patients treated with EVT. In addition, cervical ICA stenosis ≥99% (including occlusion) was independently associated with procedural duration ≥60 minutes. Although extracranial vascular characteristics increased model performance compared to baseline characteristics, this difference was only significant for procedural duration. Also, performance of the prediction models was only moderate for both procedural duration and non-successful revascularization.

Our study confirms the associations between tortuosity of the cervical ICA and long procedural duration shown by others.^5,6^ Moreover, other studies showed associations for aortic arch elongation and aortic variants,^5–8^ which were not found in our population. This difference might be due to the relative low prevalence of severe aortic arch elongation (17%) compared with two other studies (30%–39%),^7,8^ and the relatively low prevalence of aortic variant C (11%) in our study.^7^

Tortuosity of the cervical ICA was also significantly associated with non-successful revascularization, which is consistent with previous studies.^7,23^ The final prediction model for non-successful revascularization included acute take-off angle, aortic variant, and IA/CCA tortuosity, indicating an association that is consistent with previous research.^7,8^ In addition, our model also showed an independent association with tortuosity and stenosis > 99% of the cervical ICA. However, including these factors did not increase model performance and multivariate effects were only small and non-significant. One reason that we did not find a similar effect of arch elongation compared with other studies^7,8^ might have been the relative low prevalence of severe aortic arch elongation (17%) in our study. In addition, other studies were small,^5-7^ while we analyzed a large and unselected multicenter cohort. Recent studies also showed moderate performance of prediction models for revascularization after EVT,^24,25^ and our study indicates that there is no improvement after adding extracranial vascular anatomical characteristics.

Performance of our prediction models for both procedural duration and non-successful revascularization was only moderate. This might, partially, be explained by the restriction of our model to variables available prior to the start of treatment. Periprocedural factors, including the number of passes required to retrieve the thrombus, occurrence of periprocedural complications (e.g., distal emboli in other vascular territories), and thrombus characteristics are also related to procedural duration and non-successful revascularization and were not taken into account.^26–29^

Limitations of our study are the exclusion of a substantial number of patients, mainly due to incomplete depiction of the aortic arch with CTA because imaging of the aortic arch is inconsistently performed in the diagnostic workup in different hospitals. We expect this to be a random selection. In addition, we excluded patients with failed occlusion access via the transfemoral approach for the analysis of procedural duration. This exclusion possibly resulted in selection bias as this is likely related to difficult vascular anatomy. However, including these patients would have led to a higher number of patients with inconsistently shorter procedural duration. Also, model performance is likely an overestimation of the true performance, since we did not validate our prediction models.

Also, tortuosity is difficult to quantify. An additional limitation of our study is therefore that, although we introduced simple cut-off values to measure vessel tortuosity, the measurement of this parameter in routine clinical practice will still be a challenge. So far, preliminary work showed that a semi-automated method can be used to quantify tortuosity of the aortic arch and supra-aortic arteries on CTA.^30^ This technique could be useful for the identification of patients with difficult vascular anatomy but needs further development. In addition, the associations between vascular characteristics and procedural outcomes may be different for patients treated via a transradial approach. Increasing evidence suggest that EVT via the transradial approach is a safe alternative to the transfemoral approach.^31^ In both approaches, similar vascular characteristics are associated with revascularization success and increased procedural duration.^32^ In addition, patients with specific characteristics, especially difficult arch anatomy, might be better treated with a transradial approach,^33,34^ but this requires further research.

Also, we did not include experience of the interventionalist and, although patients were treated according to standard clinical practice, neurointerventional experience and equipment have improved over time. Whether intracranial anatomical characteristics (e.g., diameter of the proximal middle cerebral artery) are associated with successful revascularization was outside the scope of our analyses.^35^ Finally, we limited our analyses to the aorta, supra-aortic and cervical vessels that are visible on pre-intervention CTA. Challenging vascular anatomy in the descending aorta and iliac arteries may significantly decrease trackability of the catheter and is probably associated with revascularization and procedural duration. However, these vessels were not included in the field-of-view of the CTA scans. Hard or resistant occlusions can be another reason for unsuccessful revascularization,^36^ but whether characteristics on pre-intervention CT, such as hyperdense vessel sign, are related to treatment success is not clear^37^ and thrombus density measures are only recently improved for use in clinical practice.^38^ Both factors were therefore outside the scope of our analyses.

Strengths of our study include the use of extracranial vascular characteristics available prior to EVT on pre-intervention CTA. Also, we simplified the measured characteristics by choosing binary cut-off values, providing interventionalists with an easy and quick method to identify difficult anatomical characteristics prior to the start of EVT. In addition, results are likely generalizable to other LVO populations as data were derived from a large multicenter cohort of patients treated according to standard clinical practice with varying imaging and treatment protocols. Moreover, the large number of patients provided us with sufficient statistical power to assess the associations of all vascular characteristics from the aorta until the skull base with both procedural duration and non-successful revascularization.

## Conclusion

Our study showed that extracranial vascular characteristics had additional prognostic value compared with baseline characteristics for procedural duration but not for non-successful revascularization. Moreover, performance of both prediction models was limited in patients with anterior cerebral circulation LVO treated with EVT.

## Supplemental Material

sj-pdf-1-eso-10.1177_23969873211067662 – Supplemental Material for The prognostic value of extracranial vascular characteristics on procedural duration and revascularization success in endovascularly treated acute ischemic stroke patients Click here for additional data file. Supplemental Material, sj-pdf-1-eso-10.1177_23969873211067662 for The prognostic value of extracranial vascular characteristics on procedural duration and revascularization success in endovascularly treated acute ischemic stroke patients by Ghislaine Holswilder, Maaike PME Stuart, Tine Dompeling, Nyika D Kruyt, Jelle J Goeman, Aad van der Lugt, Wouter J Schonewille, Geert J Lycklama à Nijeholt, Charles BLM Majoie, Lonneke SF Yo, Frederick JA Meijer, Henk A Marquering, Marieke JH Wermer, Marianne AA van Walderveen, and on behalf of the MR CLEAN Registry investigators in European Stroke Journal
