# Supplementary material for: The prognostic value of extracranial vascular characteristics on procedural duration and revascularization success in endovascularly treated acute ischemic stroke patients
Source: Eur Stroke J. 2022 Feb 8;7(1):48–56. doi: 10.1177/23969873211067662 (PMC8921792; doi:10.1177/23969873211067662)
Supplement: sj-pdf-1-eso-10.1177_23969873211067662 – Supplemental Material for The prognostic value of extracranial vascular characteristics on procedural duration and revascularization success in endovascularly treated acute ischemic stroke patients [file sj-pdf-1-eso-10.1177_23969873211067662.pdf]

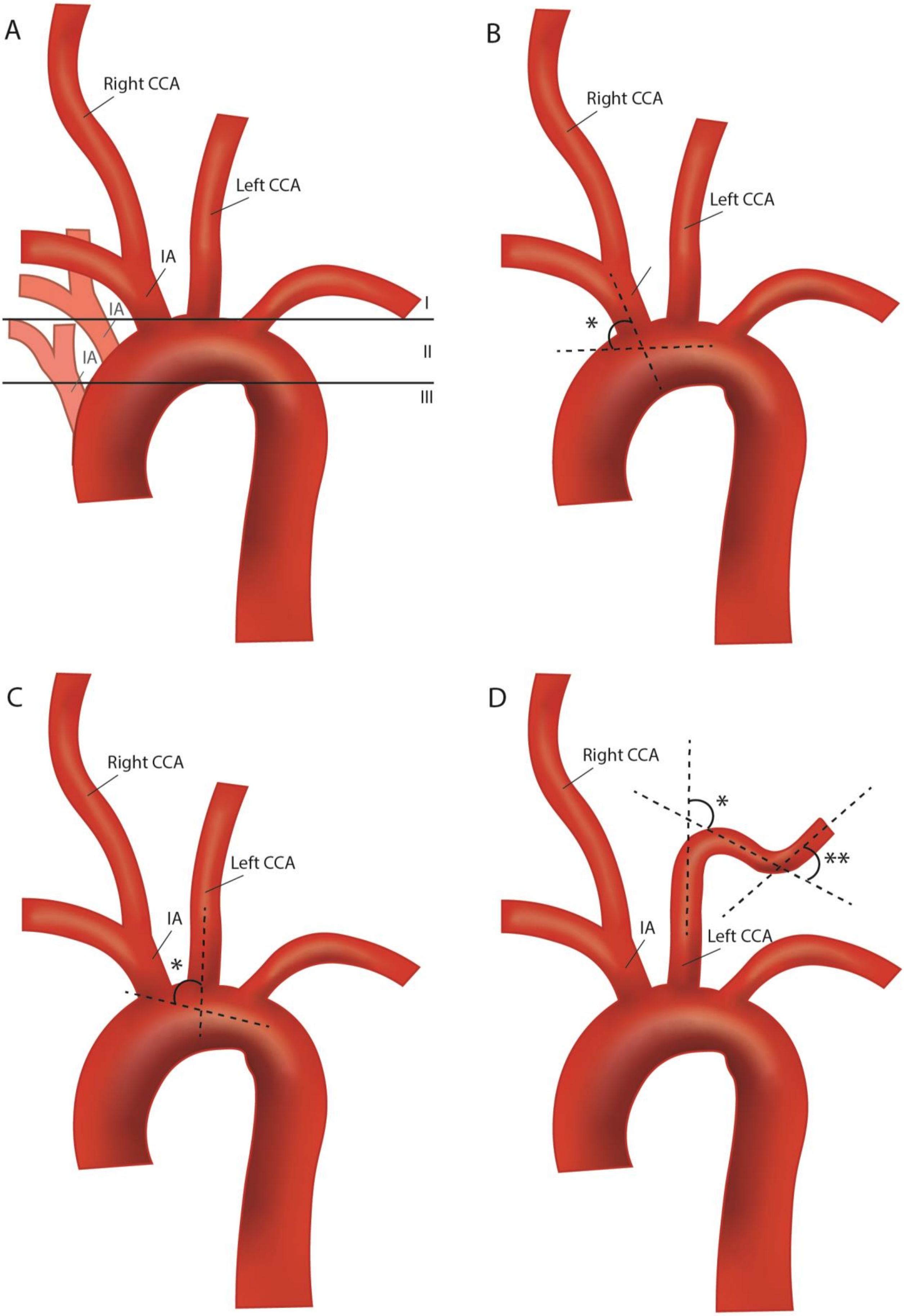
A
Right CCA
Left CCA
IA
IA
IA
I
II
III
B
Right CCA
Left CCA
*
C
Right CCA
Left CCA
IA
*
D
Right CCA
*
**
IA
Left CCA

Supplementary figure 1: Examples of measurements of aortic arch elongation, take-off angle of supra-aortic vessels and tortuosity of supra-aortic vessels on graphic interpretations of the aortic arch and supra-aortic vessels.

CCA – common carotid arteries; IA – innominate artery.

A) Graphic example of the division of aortic arch elongation into three types. On an adjusted projection showing all supra-aortic arteries simultaneously, lines are drawn on the most cranial point of the outer and inner curvatures of the aortic arch. In a type I aortic arch, the origin of all supra-aortic arteries is seen on or above the upper line, as represented by the dark red colored IA in the graphic example. The light red colored IA's are graphic examples of, respectively, a type II and type III arch. In type II, at least one of the supra-aortic arteries arises completely between the outer and inner curvature of the aortic arch. In type III, at least one supra-aortic artery arises either partially or completely below the level of the inner curvature of the arch. **B)** Graphic example of measurement of the take-off angle from the aortic arch to the IA and **C)** the take-off angle from the aortic arch to the left CCA. For both take-off angles, an adjusted projection is chosen that shows the take-off angle maximally. A first line is drawn along the center of the aortic arch, distal to the origin of the IA or left CCA, and a second line in the center of the IA or left CCA, respectively. The angle is measured at the asterisk and represents the deviation from the first line to the second line. **D)** Graphic example of the measurement of two angles in the left CCA. Measurements are performed on double oblique multiplanar reconstruction images. For each individual angle, adjusted oblique projections are chosen in such a way that the angle is observed maximally. A line is drawn in the center and along the course of the vessel segment proximal to the turning point of the angle, and a second line is drawn in the center of the vessel segment distal to the angle. The first angle indicated by one asterisk, represents an angle of ≥90°, and the second angle indicated by two asterisks, an angle of <90°.

Supplementary table 1: Variables used for multiple imputation with number of missing values and percentage of total number of patients (N=887).

**Variables**	**Missing values (%)**
Age	0 (0%)
Sex	0 (0%)
Previous stroke	6 (0.7%)
Diabetes mellitus	7 (0.8%)
Hypertension	13 (1.5%)
Atrial Fibrillation	15 (1.7%)
Systolic blood pressure	27 (3.0%)
Pre-stroke mRS	16 (1.8%)
NIHSS at baseline	21 (2.4%)
NIHSS at 24-48h follow-up	93 (10.5%)
Location of occlusion	13 (1.5%)
Pre-intervention eTICI	91 (10.3%)
Collateral score	34 (3.8%)
ASPECTS	17 (1.9%)
CBS	125 (14.1%)
IVT administered	0 (0%)
Symptom onset to groin puncture	0 (0%)
Procedural duration	89 (10.0%)
mRS after 3 months	73 (8.2%)
Post-EVT eTICI	10 (1.1%)
Failed transfemoral approach	0 (0%)
Aortic Arch Elongation	2 (0.2%)
Take-off angle	9 (1.0%)
Aortic Variants	5 (0.6%)
Number of angles in IA/CCA ≥90°	28 (3.2%)
Number of angles in ICA ≥90°	182 (20.5%)
Atherosclerosis in the origin of the IA	49 (5.5%)
Atherosclerosis in the origin of the CCA	82 (9.2%)
ICA presence of atherosclerosis	72 (8.1%)
ICA stenosis by NASCET	117 (13.2%)
intracranial ICA atherosclerosis (including stenosis)	222 (25.0%)

ASPECTS – Alberta Stroke Program Early CT Score; CBS – clot burden score; CCA – common carotid arteries; eTICI - extended Thrombolysis in Cerebral Infarction; EVT – endovascular treatment; IA – innominate artery; ICA – internal carotid artery; IVT – intravenous thrombolysis; mRS – modified Rankin Scale; NASCET – North American Symptomatic Carotid Endarterectomy Trial; NIHSS – National Institutes of Health Stroke Scale.

Supplementary table 2. Baseline and outcome characteristics of inclusions (N = 887) and exclusions (N = 601).

**Patient characteristics**	Inclusion N = 887	Exclusion N = 601	p-value
Age (mean, SD)	68.7 (14.4)	68.5 (14.5)	0.580
Men (n, %)	461 (52%)	333 (55%)	0.193
Previous stroke (n, %)	159 (18%)	90 (15%)	0.131
Diabetes mellitus (n, %)	136 (15%)	119 (20%)	**0.027**
Hypertension (n, %)	446 (51%)	299 (50%)	0.770
Atrial Fibrillation (n, %)	202 (23%)	125 (21%)	0.338
Pre-mRS ≥1 (n, %)	286 (33%)	184 (31%)	0.516
NIHSS at baseline (median, IQR)	16 (12 – 20)	16 (11 – 20)	0.748
**Radiological characteristics**			
Pre-intervention eTICI ≥1 (n, %)[a]	83 (10%)	160 (29%)	**0.000**
Collateral score ≥50% (n, %)	512 (60%)	311 (59%)	0.675
ASPECTS ≤7 (n, %)	256 (29%)	178 (32%)	0.689
CBS ≤7 (n, %)	548 (72%)	333 (70%)	0.475
**Intervention characteristics**			
IVT administered (n, %)	683 (77%)	478 (80%)	**0.044**
EVT under general anesthesia (n, %)	231 (27%)	148 (27%)	0.915
Symptom onset to groin puncture, min (median, IQR)	210 (159 – 265)	205 (160 – 268)	0.613
**Outcome characteristics**			
Procedural duration, min (median, IQR)[b]	65 (45 – 90)	60 (35 – 90)	**0.001**
eTICI[a]			**0.029**
0	171 (19%)	93 (16%)	
1	32 (4%)	16 (3%)	
2A	192 (22%)	114 (19%)	
2B	162 (18%)	109 (18%)	
2C	85 (10%)	53 (9%)	
3	235 (27%)	205 (35%)	
mRS after 3 months 3-6	504 (62%)	342 (62%)	0.813

ASPECTS – Alberta Stroke Program Early CT Score; CBS – clot burden score; eTICI - extended Thrombolysis in Cerebral Infarction; EVT – endovascular treatment; IQR – interquartile range; IVT – intravenous thrombolysis; mRS – modified Rankin Scale; SD – standard deviation.

P-values were calculated using Pearson Chi-Squared test for categorical variables and Mann-Whitney U test for continuous variables.

[a]Pre-intervention eTICI and outcome variable eTICI are related to the exclusion criteria, as the largest number (n = 119) of exclusions was due to spontaneous reperfusion occurring prior to EVT and this, in general, results in a pre- and postintervention eTICIs ≥1.

[b]Procedural duration is related to the exclusion criteria of a patient, as patients without full EVT procedure were excluded, leading to an average shorter duration in the excluded group.

Supplementary table 3. Number of patients with procedural duration ≥60 minutes (n=828) and non-successful revascularization (eTICI 0-2A) (n=887) in the presence versus absence of extracranial vascular characteristics.

	**Duration ≥60 min. with Characteristic Present (N) (%)**	**Duration ≥60 min. with Characteristic Absent (N) (%)**	**eTICI 0–2A with Characteristic Present (N) (%)**	**eTICI 0–2A with Characteristic Absent (N) (%)**
**Aortic Arch Elongation**				
Type II vs. I	297/489 (61%)	80/137 (58%)	241/554 (44%)	70/154 (46%)
Type III vs. I	78/126 (62%)	80/137 (58%)	84/167 (50%)	70/154 (46%)
Take-off angle ≥135°	38/57 (67%)	413/690 (60%)	36/69 (53%)	356/799 (45%)
**Aortic Variants**				
Variant B vs. A	60/99 (61%)	341/568 (60%)	48/120 (40%)	287/648 (44%)
Variant C vs A	52/84 (62%)	341/568 (60%)	56/104 (54%)	287/648 (44%)
**Tortuosity right IA and CCA, or left CCA[a, b]**				
Presence of ≥1 angles ≥90°	175/279 (63%)	266/453 (59%)	165/335 (49%)	216/515 (42%)
Presence of ≥2 angles ≥90°	65/104 (63%)	376/628 (60%)	63/124 (51%)	318/726 (44%)
**Tortuosity cervical ICA[b]**				
Presence of ≥1 angles ≥90°	206/323 (64%)	131/282 (46%)	183/371 (49%)	124/325 (38%)
Presence of ≥2 angles ≥90°	107/154 (69%)	230/451 (51%)	92/183 (50%)	215/513 (42%)
**Atherosclerosis**				
IA/CCA origin stenosis ≥50%	12/12 (100%)	395/674 (59%)	11/14 (79%)	342/778 (44%)
ICA stenosis ≥99%	54/65 (83%)	336/596 (56%)	44/86 (51%)	300/675 (44%)
Intracranial stenosis ≥50%	50/89 (56%)	265/481 (55%)	54/109 (50%)	231/547 (42%)

CCA – common carotid arteries; eTICI - extended Thrombolysis in Cerebral Infarction; IA – innominate artery; ICA – internal carotid artery.

Numbers might not add up due to missing values.

[a]The IA and left or right CCA were measured ipsilateral to the side of the intracranial vessel occlusion.

[b]Presence of ≥1 angles ≥90° was compared with no angle ≥90°; presence of ≥2 angles ≥90° was compared with no or 1 angle ≥90°.

Supplementary table 4. Regression coefficients of characteristics included in the final model for prediction of procedural duration ≥60 minutes (N=828).

**Characteristic**	Regression coefficient (β)	*P* value
Age	-0.005	0.334
Hypertension	0.273	0.113
CBS		
0	reference	
1	0.002	0.998
2	-0.166	0.808
3	-0.488	0.469
4	-0.778	0.150
5	-0.718	0.222
6	-0.883	0.086
7	-0.950	0.087
8	-0.981	0.061
9	-0.360	0.517
10	-1.191	0.152
Tortuosity cervical ICA[a]	0.676	0.000
ICA stenosis ≥99%	1.162	0.001
**Intercept**	0.909	0.142

CBS – clot burden score; ICA – internal carotid artery.

[a]Presence of ≥1 angles ≥90°.

Supplementary table 5. Regression coefficients of characteristics included in the final model for prediction of non-successful revascularization (eTICI 0–2A) (N=887).

**Characteristic**	Regression coefficient (β)	*P* value
Age	0.000	0.992
Hypertension	-0.057	0.714
Collateral score		
Absent collaterals	reference	
Filling ≤50%	-0.167	0.579
Filling >50% but <100%	-0.546	0.068
Filling 100%	-0.375	0.253
CBS		
0	reference	
1	0.168	0.726
2	0.293	0.580
3	0.072	0.885
4	-0.313	0.485
5	0.124	0.803
6	-0.194	0.643
7	0.192	0.696
8	0.033	0.940
9	0.121	0.789
10	0.331	0.664
Pre-intervention eTICI		
0	reference	
1	0.070	0.863
2A	-0.033	0.947
2B-3	-0.035	0.949
IVT	-0.165	0.341
Take-off angle ≥135°	0.537	0.065
Aortic Variants		
Variant A	reference	
Variant B	-0.292	0.221
Variant C	0.450	0.045
Tortuosity right IA and CCA, or left CCA[a]	0.217	0.184
Tortuosity cervical ICA[b]	0.405	0.009
IA/CCA origin stenosis ≥50%	1.217	0.108
ICA stenosis ≥99%	0.324	0.196
**Intercept**	-0.152	0.797

CBS – clot burden score; CCA – common carotid arteries; eTICI - extended Thrombolysis in Cerebral Infarction; IA – innominate artery; ICA – internal carotid artery; IVT – intravenous thrombolysis.

[a]The IA and left or right CCA were measured ipsilateral to the side of the intracranial vessel occlusion.

[b]Presence of ≥1 angles ≥90°.

Supplementary acknowledgements.

MR CLEAN Registry Investigators – group authors

Executive committee

Diederik W.J. Dippel[1];Aad van der Lugt[2];Charles B.L.M. Majoie[3];Yvo B.W.E.M. Roos[4];Robert J. van Oostenbrugge[5];Wim H. van Zwam[6];Jelis Boiten[14];Jan Albert Vos[8]

Study coordinators

Ivo G.H. Jansen[3]; Maxim J.H.L. Mulder[1,2];Robert- Jan B. Goldhoorn[5,6];Kars C.J. Compagne[2];Manon Kappelhof[3]

Local principal investigators

Wouter J. Schonewille[7];Jan Albert Vos[8];Charles B.L.M. Majoie[3];Jonathan M. Coutinho[4];Marieke J.H. Wermer[9];Marianne A.A. van Walderveen[10];Julie Staals[5];Wim H. van Zwam[6];Jeannette Hofmeijer[11];Jasper M. Martens[12];Geert J. Lycklama à Nijeholt[13];Jelis Boiten[14];Bob Roozenbeek[1];Bart J. Emmer[2];Sebastiaan F. de Bruijn[15];Lukas C. van Dijk[16];H. Bart van der Worp[17] ;Rob H. Lo[18];Ewoud J. van Dijk[19];Hieronymus D. Boogaarts[20];Paul L.M. de Kort[21]; Julia van Tuijl[21] ;Jo J.P. Peluso[26];Jan S.P. van den Berg[22];Boudewijn A.A.M. van Hasselt[23];Leo A.M. Aerden[24];René J. Dallinga[25];Maarten Uyttenboogaart[28];Omid Eshghi[29]; Reinoud P.H. Bokkers[29];Tobien H.C.M.L. Schreuder[30];Roel J.J. Heijboer[31];Koos Keizer[32];Lonneke S.F. Yo[33];Heleen M. den Hertog[22];Emiel J.C. Sturm[35]

Imaging assessment committee

Charles B.L.M. Majoie[3](chair);Wim H. van Zwam[6];Aad van der Lugt[2];Geert J. Lycklama à Nijeholt[13];Marianne A.A. van Walderveen[10];Marieke E.S. Sprengers[3];Sjoerd F.M. Jenniskens[27];René van den Berg[3];Albert J. Yoo[37];Ludo F.M. Beenen[3];Alida A. Postma[6];Stefan D. Roosendaal[3];Bas F.W. van der Kallen[13];Ido R. van den Wijngaard[13];Adriaan C.G.M. van Es[2];Bart J. Emmer[2,3];Jasper M. Martens[12]; Lonneke S.F. Yo[33];Jan Albert Vos[8]; Joost Bot[36], Pieter-Jan van Doormaal[2].

Writing committee

Diederik W.J. Dippel[1](chair);Aad van der Lugt[2];Charles B.L.M. Majoie[3];Yvo B.W.E.M. Roos[4];Robert J. van Oostenbrugge[5];Wim H. van Zwam[6];Geert J. Lycklama à Nijeholt[13];Jelis Boiten[14];Jan Albert Vos[8];Wouter J. Schonewille[7];Jeannette Hofmeijer[11];Jasper M. Martens[12];H. Bart van der Worp[17];Rob H. Lo[18]

Adverse event committee

Robert J. van Oostenbrugge[5](chair);Jeannette Hofmeijer[11];H. Zwenneke Flach[23]

Trial methodologist

Hester F. Lingsma[38]

Research nurses / local trial coordinators

Naziha el Ghannouti[1];Martin Sterrenberg[1];Corina Puppels[7];Wilma Pellikaan[7];Rita Sprengers[4];Marjan Elfrink[11];Joke de Meris[14];Tamara Vermeulen[14];Annet Geerlings[19];Gina van Vemde[22];Tiny Simons[30];Cathelijn van Rijswijk[21];Gert Messchendorp[28];Hester Bongenaar[32];Karin Bodde[24];Sandra Kleijn[34];Jasmijn Lodico[34]; Hanneke Droste[34];M. Wollaert[5];D. Jeurrissen[5];Ernas Bos[9];Yvonne Drabbe[15];Nicoline Aaldering[11];Berber Zweedijk[17];Mostafa Khalilzada[15].

PhD / Medical students:

Esmee Venema[38]; Vicky Chalos[1,38]; Ralph R. Geuskens[3]; Tim van Straaten[19]; Saliha Ergezen[1]; Roger

R.M. Harmsma[1]; Daan Muijres[1]; Anouk de Jong[1]; Wouter Hinsenveld[5,6];Olvert A. Berkhemer[1,3,6];Anna M.M. Boers[3,39]; J. Huguet[3];P.F.C. Groot[3];Marieke A. Mens[3];Katinka R. van Kranendonk[3];Kilian M. Treurniet[3];Manon L. Tolhuijsen[3];Heitor Alves[3].

List of affiliations
Department of Neurology[1], Radiology[2], Public Health[38], Erasmus MC University Medical Center;
Department of Radiology and Nuclear Medicine[3], Neurology[4], Biomedical Engineering & Physics[39], Amsterdam UMC, University of Amsterdam, Amsterdam;
Department of Neurology[5], Radiology[6], Maastricht University Medical Center and Cardiovascular Research Institute Maastricht (CARIM);
Department of Neurology[7], Radiology[8], Sint Antonius Hospital, Nieuwegein;
Department of Neurology[9], Radiology[10], Leiden University Medical Center;
Department of Neurology[11], Radiology[12], Rijnstate Hospital, Arnhem;
Department of Radiology[13], Neurology[14], Haaglanden MC, the Hague;
Department of Neurology[15], Radiology[16], HAGA Hospital, the Hague;
Department of Neurology[17], Radiology[18], University Medical Center Utrecht;
Department of Neurology[19], Neurosurgery[20], Radiology[27], Radboud University Medical Center, Nijmegen;
Department of Neurology[21], Radiology[26], Elisabeth-TweeSteden ziekenhuis, Tilburg;
Department of Neurology[22], Radiology[23], Isala Klinieken, Zwolle;
Department of Neurology[24], Radiology[25], Reinier de Graaf Gasthuis, Delft;
Department of Neurology[28], Radiology[29], University Medical Center Groningen;
Department of Neurology[30], Radiology[31], Atrium Medical Center, Heerlen;
Department of Neurology[32], Radiology[33], Catharina Hospital, Eindhoven;
Department of Neurology[34], Radiology[35], Medical Spectrum Twente, Enschede;
Department of Radiology[36], Amsterdam UMC, Vrije Universiteit van Amsterdam, Amsterdam;
Department of Radiology[37], Texas Stroke Institute, Texas, United States of America.
